# Exploring the Impact of Extended Amino Acid Supplementation on Body Composition and Psychopathology in Patients with Eating Disorders Undergoing Psycho-Nutritional Rehabilitation

**DOI:** 10.2174/0118715303310253241210080238

**Published:** 2025-01-03

**Authors:** Michela Napolillo, Laura Dalla Ragione, Giulia De Iaco, Tania Mococci, Roberto Sacco, Simonetta Marucci

**Affiliations:** 1 Independent Researcher (Nutritional Biologist), Rome, Italy;; 2 Department of Food Science and Human Nutrition, Campus Biomedico, University of Rome, Rome, Italy;; 3 Independent Researcher (Nutritional Biologist), Spoleto, Perugia, Italy;; 4 Department of Psychology, Azienda Ospedaliera of Perugia, Perugia, Italy;; 5 Department of Child Neuropsychiatry, Campus Biomedico, University of Rome, Rome, Italy

**Keywords:** Eating disorders, protein-energy malnutrition, amino acid supplementation, body composition, psychometric tests, bioelectrical impedance analysis

## Abstract

**Background:**

Eating disorders are frequently linked to protein-energy malnutrition, resulting from chronic energy intake reduction, and leading to reduced body weight and altered body composition. A nutritional approach based on the supplementation with a mixture of essential amino acids has proven effective in conditions characterized by reduced dietary intake and increased degradation of endogenous proteins and might also contribute to correct the amino acid deficiency possibly involved in the pathogenesis of eating disorders.

**Objective:**

This study aimed to explore the effects of amino acid supplementation in conjunction with a standard psycho-nutritional rehabilitation program on body composition, symptoms and psychopathology status in 30 female patients with eating disorders hospitalized at the Residenza Palazzo Francisci in Todi, Italy.

**Methods:**

Enrolled patients were divided in two groups receiving amino acid supplementation for 45 and 90 days, respectively. Each patient underwent bioelectrical impedance analysis at baseline, after 45 days and after 90 days. Validated psychometric tests were administered at study entry and study end to all enrolled patients.

**Results:**

Results from bioelectrical impedance analysis suggest that amino acid supplementation contributes to restoring nutritional and metabolic balance, with effects proportional to the duration of administration. Conversely, interpreting the effects on psychopathologic state and symptoms was challenging, probably due to the small sample size and short observation period.

**Conclusion:**

Prolonged amino acid supplementation may play a role in the management of patients with eating disorders, favoring the correction of the catabolic state and possibly contributing to improvement of the psychopathologic state.

## 1 INTRODUCTION

Nutritional status is the result of three strictly correlated variables: body composition, energy balance and body function. Eating disorders (EDs) are a primary cause of altered nutritional status, encompassing severe malnutrition in nervous anorexia (NA) to severe obesity and metabolic syndrome in nervous bulimia (NB) and binge eating disorder (BED). In individuals with NA, prolonged dietary restriction and subsequent malnutrition lead to several complications affecting cardiovascular, respiratory, gastrointestinal, endocrine, and other systems [1-3]. Dietary restriction is often associated with purging (self-induced vomiting and use of laxatives) and physical hyperactivity [4-7]. Organic complications of NB mainly depend on binge eating and purging rather than on dietary restriction.

The organic consequences of impaired nutritional status associated with EDs may affect every organ, depending on the patient’s characteristics such as gender, age, and comorbidities. A typical condition associated with EDs is Protein-Energy Malnutrition (PEM) due to prolonged hypo-nutrition [8]. PEM is characterized by a severe decrease of body weight, alteration of body composition (progressive reduction of fat and lean mass, due to the forced use of protein stores as a substrate for gluconeogenesis) and the induction of adaptive metabolic/hormonal mechanisms, such as a reduction of basal metabolism. For this reason, the assessment of body composition is crucial in individuals with EDs and PEM.

Bioelectrical impedance analysis (BIA), an indirect technique to assess body composition [9], is widely employed in studying changes in various body districts under PEM conditions, being not invasive and easy to use. BIA is based on the study of resistance (impedance) opposed by tissues to the application of alternate current with very low intensity (400 µÅ) and very high frequency (50 kHz). Impedance (Z) depends on the resistance (Rz) and reactance (Xc) of the tissue: Rz, which is the tissue’s capacity to resist to the transmission of an electric current, is inversely related to the amount of water contained in the tissue. On the other hand, Xc, which is the capacity of the tissue to act as a capacitor, is directly related to cell concentration and represents an indirect measure of active cellular membranes. BIA is, therefore, particularly useful to assess body water content and its distribution in intra- and extracellular spaces, providing an estimation of hydration status. Indeed, the role of BIA assessment in ED management goes beyond the evaluation of nutritional status, potentially contributing to improve disease awareness and reduce the patient’s focus on controlling body weight. The physician’s reporting of the examination is essential to convince patients to consider weight not merely as a number, but as the result of many factors contributing to health status. In this perspective, weight changes can be accepted as an expression of the interaction between different body compartments, rather than denied or considered as a failure. This may contribute to increase the patient’s motivation and adherence to therapy.

Amino acids (AAs) play an essential role in maintaining the structural and functional integrity of tissues and organs, and their availability in adequate amount is crucial to ensure protein synthesis. Essential AAs, which cannot be produced endogenously, must be introduced with food. The importance of their intake lies in preventing protein catabolism and loss of non-essential AAs [10, 11]. In physiologic conditions, essential AAs include valine, isoleucine, leucine, phenylalanine, threonine, methionine, lysine, histidine and tryptophan. In catabolic states (infancy, malnutrition), other AA, like glutamine, arginin, cystein and tyrosine, become essential as well [12-14].

In physiologic conditions, protein turnover is maintained by the balance between protein synthesis and disposition. These processes are coordinated and regulated by distinct mechanisms [15]. In catabolic conditions, the extensive degradation of muscle proteins leads to a reduction of both muscle mass and function [16]. Increased muscular protein degradation is generally associated to a poor dietary intake of AAs, especially essential AAs, further enhancing muscle degradation in the attempt to compensate for the negative effect of inadequate protein intake on tissue growth [17, 18]. A nutritional approach based on the supplementation with a mixture of essential AAs has proven effective in conditions characterized by reduced dietary intake and increased degradation of endogenous proteins. This approach results in increased mitochondrial number and function, improved muscle metabolism and higher efficiency in energy production [19]. Supplemented essential AAs also promote muscular tissue growth by counteracting proteolysis and improving the synthesis of muscle and plasma proteins [20]. In humans, essential AA supplementation has shown positive effects on fat free mass (FFM), muscular strength, exercise endurance and physical performance in both healthy subjects and in various conditions associated with impaired nutritional and metabolic state [21], such as chronic heart failure and chronic obstructive pulmonary disease [22-27], hemodialysis [28-30], HIV [31], cancer cachexia [32], sarcopenia [33] and Type 2 diabetes [34, 35].

This provides the rationale for the potential usefulness of essential AA supplementation in patients with EDs, who frequently present an altered nutritional status associated with PEM. Moreover, their deficiency might be involved in the pathogenesis of EDs, as suggested by studies demonstrating that a low availability of AAs may limit the synthesis of monoaminergic neurotransmitters, such as noradrenaline, dopamine, and serotonin [36, 37] which contribute to the regulation of eating behavior [38-42].

EDs are associated with a specific form of PEM caused by chronic reduction of energy intake. In this condition, malnutrition leads to organic damage, which at the cerebral level may contribute to worsening behavioral disturbances. Psycho-Nutritional Rehabilitation (PNR) programs aim at restoring nutritional and metabolic balance thereby counteracting behavioral disturbances: however, low compliance and poor motivation may slow down the recovery process.

In principle, the use of BIA assessments and psychometric questionnaires should be useful to monitor the clinical course of the disease during the PNR and may help to increase the patient’s awareness, potentially improving adherence to treatment. On the other hand, the use of self-administered questionnaires for evaluating symptoms severity may help the patient to better understand the role of the psychological sphere in the pathogenesis of the ED. Moreover, in hypercatabolic conditions such as NA and NB, where the nutritional intervention must be hypocaloric to avoid the refeeding syndrome, the use of AA supplementation may be useful to speed up the nutritional and metabolic recovery.

Therefore, the main objective of this study was to assess the efficacy of daily supplementation (4 g twice daily) with a mixture of essential AAs (AMINOTROFIC^®^) in the first 45 days of PNR at the Residenza Palazzo Francisci in Todi (T0-T1) in terms of BIA parameters. Additionally, we aimed at evaluating the advantages of a further supplementation period at the dose of 4 g/daily in the following 45 days of PNR (T1-T2) on BIA parameters. Finally, we sought to study the correlation, if any, between body composition (as assessed by BIA) and the degree of psychopathology and severity of reported symptoms (evaluated through the SCL-90 and EDI-2 tests) at T0 (baseline) and T2 (end of study).

## 2 MATERIALS AND METHODS

### 2.1 Subjects

A total of 30 patients admitted to the Residenza Palazzo Francisci in Todi (median age 21.5 years) were enrolled in this study, all diagnosed with ED according to DSM-V. Among them, 25 patients had a diagnosis of NA, 4 of NB, and 1 of avoidant/restrictive food intake disorder (ARFID). Since no male patient was admitted at the time of study recruitment (in line with the higher prevalence of EDs in women), all included patients were female. All patients were hospitalized for the entire study duration (3 months). Patients were randomized into 2 groups: the first one (Group 1), undergoing AA supplementation at the dose of 4 g twice daily in the first 45 days (T0-T1) and at the dosage of 4 g/day in the following 45 days (T1-T2), included 14 patients (10 with NA, 3 with NB and 1 with ARFID). The second group (Group 0), undergoing AA supplementation at the dose of 4 g twice daily in the first 45 days (T0-T1) and no supplementation in the following 45 days (T1-T2), included 16 patients (15 with NA, 1 with NB). All subjects gave their written informed consent to participate in the study. The study design is shown in Fig. (**1**).

### 2.2 Treatments

The standard PNR program used at the Residenza Palazzo Francisci in Todi included an initial menu of 1700 Kcal. After two weeks, this was followed by a menu of 1960 Kcal, and after an additional two weeks, a menu of 2450 Kcal was implemented, divided in five meals throughout the day (two main meals and three snacks). During the first 45 days of hospitalization (T0-T1), all enrolled patients underwent the standard PNR associated with daily supplementation with AMINOTROFIC^®^, a mixture of essential and non-essential AAs containing L-Leucine, L-Lisine, L-Isoleucine, L-Valine, L-Threonine, L-Cystine, L-Histidine, L-Phenylalanine, L-Methionine, L-Tyrosine, L-Tryptophan, vitamin B6 and vitamin B1. This was administered at the dosage of 4 g twice daily, at 11 am and 10 pm, without food, to warrant complete absorption and anabolic support throughout the day. In the following 45 days (T1-T2), AMINOTROFIC^®^ was administered at the dosage of 4 g daily at 11 am in group 1 only, whereas supplementation was discontinued in group 0.

### 2.3 Assessments

#### 2.3.1 Bioelectrical Impedance Analysis

Each patient underwent three BIA assessments, at baseline (T0), after 45 days (T1) and after 90 days (T2) using the BIA AKERN 101 device, and the BodyGram PRO 3.0 software. Through this software, we obtained several parameters which typically define body composition:

- Phase Angle (PA): it is the ratio of Rz to Xc and serves as an indicator of the general physical status (normal range: 4-9°): high values may indicate dehydration or a high amount of intact cell membranes (as seen in athletes), whereas low values may suggest water retention or loss of functional cell membranes (as observed in the presence of PEM) [43].
- Total Body Water (TBW): it includes intracellular (ICW) and extracellular water (ECW), with values varying based on nutritional status, age and gender. Low values are typically associated with obesity, given the low water content in adipose tissue [43].
- Body Cell Mass (BCM): it represents the total volume of living cells, constituting 35-40% of body weight. BMC is the active compartment of the body, consuming oxygen for energy substrate utilization (e.g. glucose and lipids): its value, indicative of energy expenditure, protein needs and metabolic response to physiologic or pathologic stimuli, increases with physical activity and decreases with age and pathology [43].
- Fat Free Mass (FFM): it includes muscle, bones, and other non-fat tissues, composed of water (73%), proteins (20%) and minerals (7%) [43].
- Fat Mass (FM): this includes all lipids in the body, and its value (range 16-22% in men; 20-25% in women) is calculated as the difference between BW and FFM. It is reliable only when hydration status is within the normal range [43].
- Basal Metabolic Rate (BMR): it represents the energy expenditure in resting conditions, covering vital functions like respiration, circulation, digestion and nervous system activity. BMR corresponds to 45-75% of total energy expenditure [43].
- Muscle Mass (MM): it is the portion of muscle-skeletal tissue made of red and white muscle fibres, corresponding to 50% of FFM [43].
- Extra Cellular Mass (ECM): it corresponds to body tissues and fluids outside cells, including plasma, interstitial fluids, cerebrospinal fluid, articular fluids, tendons, derma, collagen, elastin, and the skeleton. Interstitial fluid (or extracellular water, ECW) is the main portion of ECM, undergoing rapid and substantial changes; it is calculated as FFM-BMC.
- ECM/BCM: it is the ratio between extracellular and intracellular spaces (normal range: 0.9-1). It is useful to monitor nutrition and hydration status in the presence of PEM: values <1 indicate dehydration, whereas values >1 indicate hyperhydration or a catabolic state.
- Na/K Exchange: it is the exchange ratio between extracellular Na and intracellular K (normal range: 0.85-0.9 in men; 0.9-1 in women). Maintenance of Na-K balance across the cellular membrane is crucial, and values on Na/K exchange >1 indicate K loss.
- Body Cell Mass Index (BCMI): it is the ratio between cellular mass and height (normal values: ≥8 in men; ≥7 in women). Together with PA and hydration status it allows to assess nutritional status: low BCMI values may indicate malnutrition (PEM), whereas high values indicate a large muscle mass (as seen in athletes).

In addition, weight and height were recorded for each patient, and the body Mass Index (BMI) was calculated as the ratio between weight in Kg and height in m^2^.

#### 2.3.2 Psychometric Tests

Validated psychometric tests, such as the Eating Disorder Inventory-2 (EDI-2) [44] and the Symptom Checklist-90 (SCL-90) [45] were administered at T0 and T2 to all enrolled patients.

### 2.4 Statistical Analysis

Matching of enrolled patients in the two groups at baseline was assesses using an unpaired T-test for both BIA parameters and psychometric scores. A paired T-test was then performed to analyze differences of BIA parameters and psychometric scores at different time intervals (T0-T1, T1-T2 and T0-T2) within each group. Correlations between body composition (as assessed by BIA) and the degree of psychopathology and severity of reported symptoms (as assessed through the SCL-90 and EDI-2 tests) at T0 and T2 were analyzed using a simple linear regression model and the calculation of Pearson coefficient. Considering the explorative nature of the study, correction for multiple comparisons was not performed. The statistical analysis was performed by SPSS software.

## 3 RESULTS

Comparison of BIA parameters and psychometric scores in the 2 groups at baseline (T0) found no differences between groups. Changes within each group of BIA parameters and psychometric scores at different time intervals (T0-T1, T1-T2 and T0-T2), and correlations between body composition and the degree of psychopathology and severity of reported symptoms at T0 and T2 are reported below.

### 3.1 BIA Parameters

**Fig. (2):**
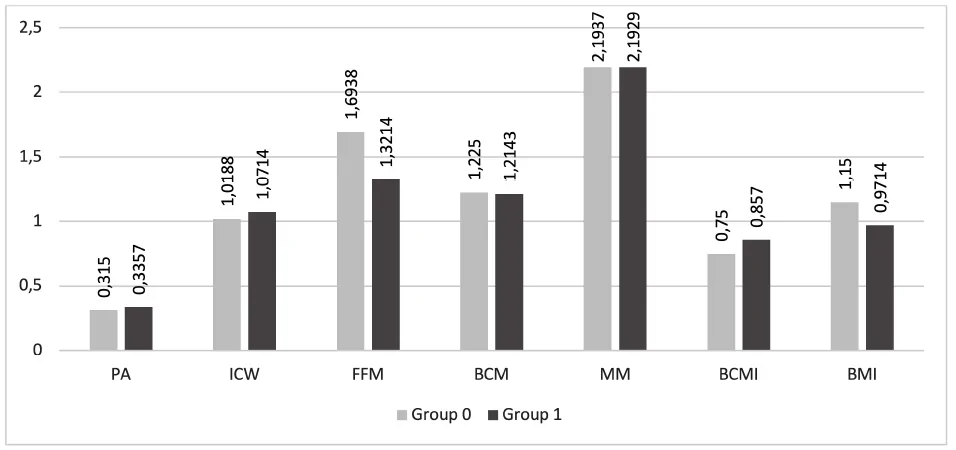
Paired differences of BIA parameters from T0 to T1 reaching statistical significance in both groups. **Abbreviations:** PA, phase angle; ICW, intracellular water; FFM, fat free mass; BCM, body cell mass; MM, muscle mass; BCMI, body cell mass index; BMI, body mass index.

T0-T1 Interval: statistically significant (*p* < 0.05) increases of PA, TBW, ICW, FFM, BCM, MM, BMR, BCMI and BMI and statistically significant (*p* < 0.05) decreases of ECM/BCM and Na/K exchange were observed in group 0. In group 1, statistically significant (*p* < 0.05) increases of PA, ICW, FFM, BCM, MM, BMR, BCMI and BMI were recorded. Considering differences of BIA parameters from T0 to T1 reaching statistical significance in both groups, similar changes were observed (Fig. **2**).

**Fig. (3):**
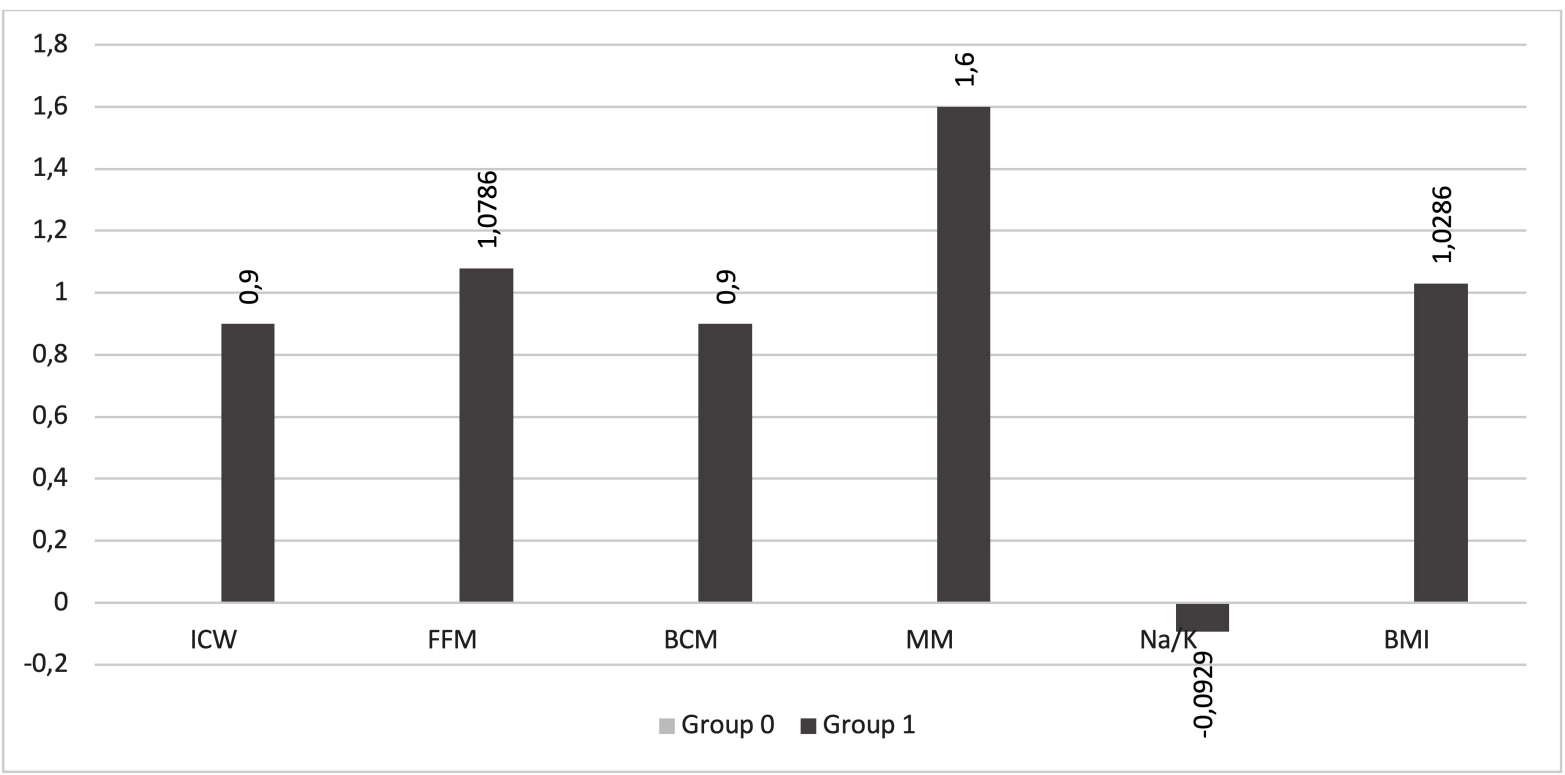
Statistically significant paired differences of BIA parameters from T1 to T2. **Abbreviations:** ICW, intracellular water; FFM, fat free mass; BCM, body cell mass; MM, muscle mass; Na/K, Na/K exchange; BMI, body mass index.

T1-T2 Interval: no statistically significant difference was observed in group 0, whereas statistically significant (*p* < 0.05) increases of ICW, FFM, BCM, MM, BMR and BMI, as well as a statistically significant (*p* < 0.05) decrease of Na/K exchange were recorded in group 1 (Fig. **3**).

**Fig. (4):**
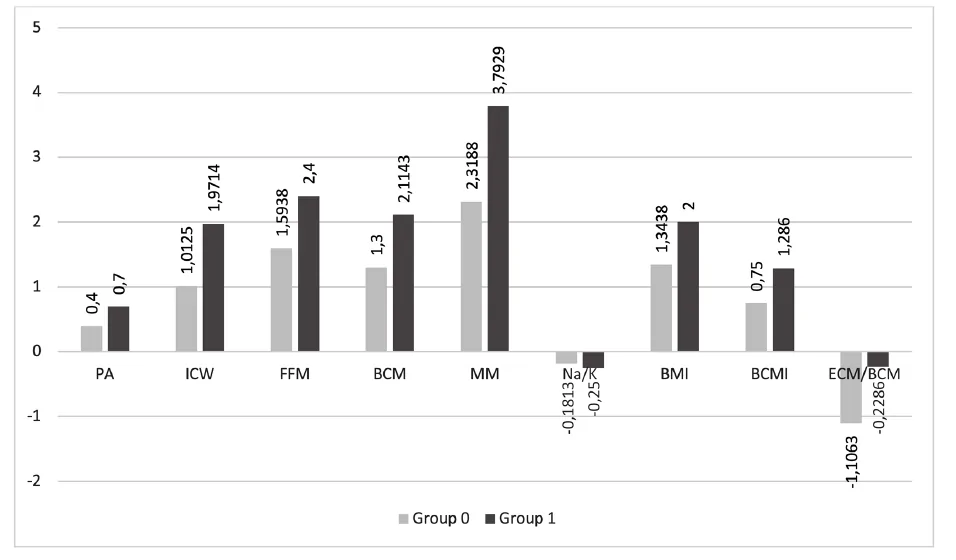
Paired differences of BIA parameters from T0 to T2 reaching statistical significance in both groups. **Abbreviations:** PA, phase angle; ICW, intracellular water; FFM, fat free mass; BCM, body cell mass; MM, muscle mass; BCMI, body cell mass index; Na/K, Na/K exchange; BMI, body mass index.

T0-T2 Interval (whole study period): statistically significant (*p* < 0.05) increases of PA, ICW, FFM, BCM, MM, BMR, BCMI and BMI, as well as statistically significant (*p* < 0.05) decreases of ECM/BCM and Na/K exchange were observed in group 0. In group 1, statistically significant (*p* < 0.05) increases of PA, TBW, ICW, FFM, BCM, MM, BMR, BCMI and BMI were recorded, accompanied by statistically significant (*p* < 0.05) decreases of ECM/BCM and Na/K exchange. Considering paired differences of BIA parameters from T0 to T2 reaching statistical significance in both groups, changes were more pronounced in group 1. For certain BIA parameters, including PA, ICW and BCMI, group 1 exhibited almost a two-fold increase in the mean paired differences compared to group 0 (Fig. **4**).

### 3.2 Psychometric Tests (T0-T2 Interval)

**Fig. (5):**
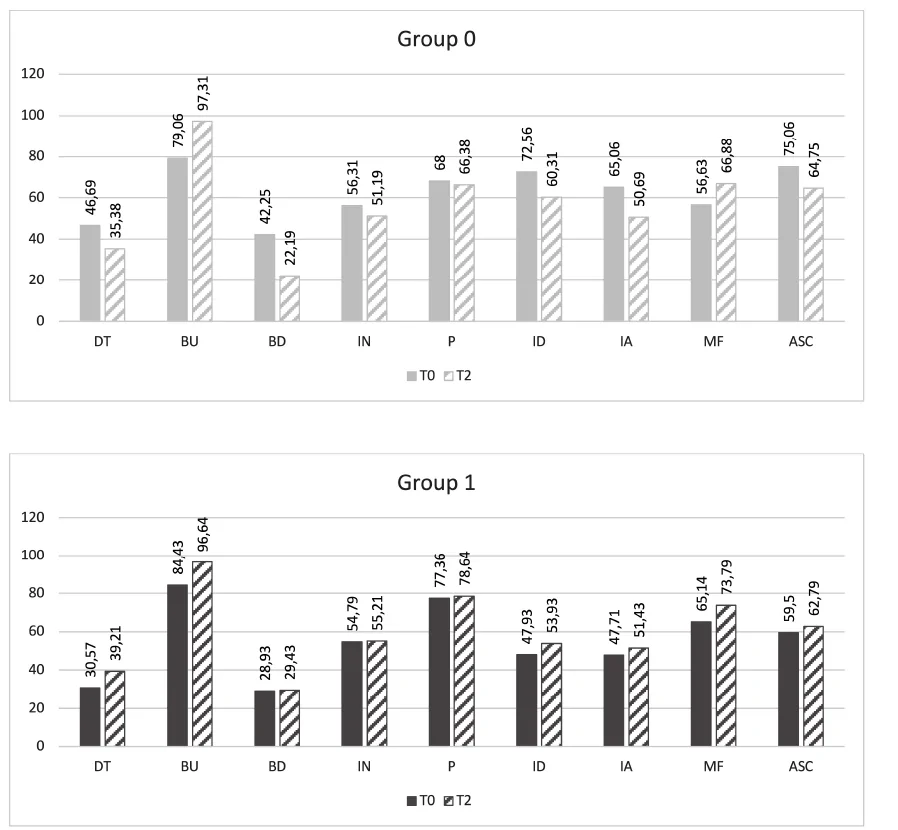
Values of EDI-2 subscales recorded in each group at the T0 and T2 timepoints (*p < 0.05). **Abbreviations:** DT, drive for thinness; BU, bulimia; BD, body dissatisfaction; IN, ineffectiveness; P, perfectionism; ID, interpersonal distrust; IA, interoceptive awareness; MF, maturity fears; ASC, ascetism.

EDI-2: in group 0, a statistically significant increase of BU subscale (relative to bulimia; *p* = 0.019) and a statistically significant decrease of BD (*p* = 0.034) and IA (*p* = 0.042) subscales (regarding body unsatisfaction and enteroceptive awareness, respectively) were observed. Conversely, non-significant increases were recorded in all subscales in group 1 (Fig. **5**).

**Fig. (6):**
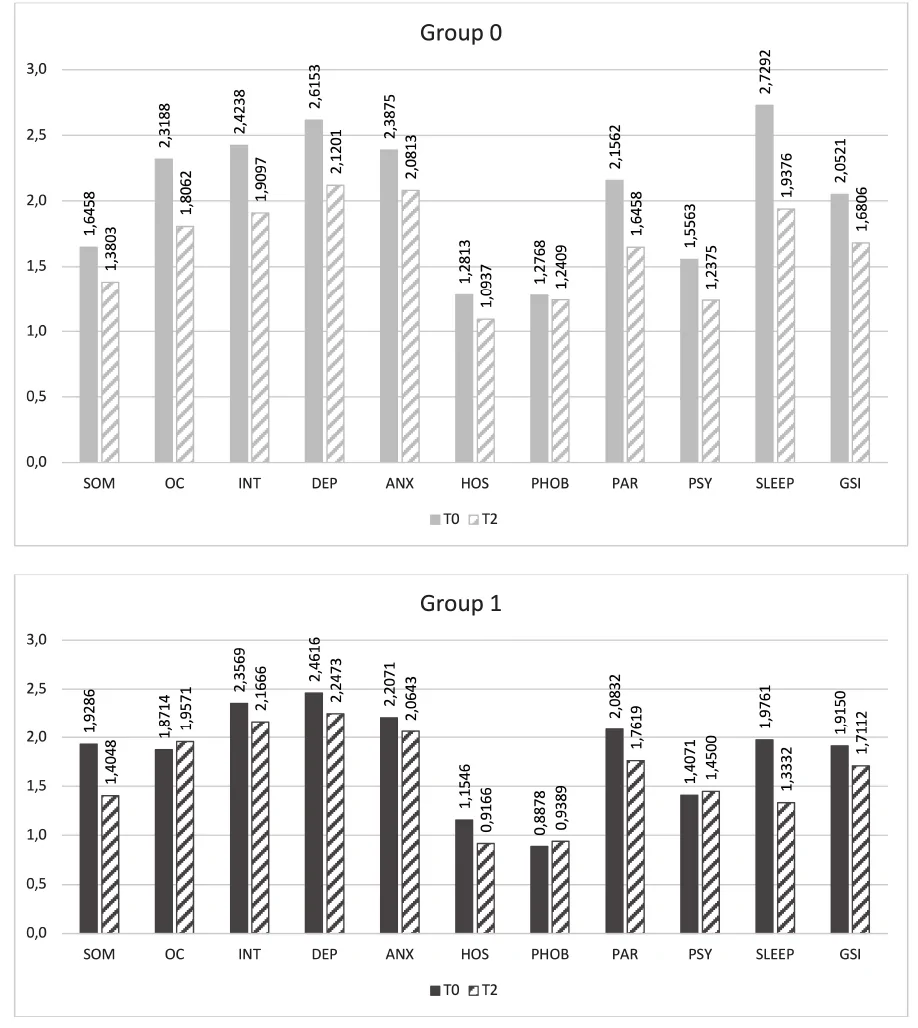
Values of SCL-90 subscales recorded in each group at the T0 and T2 timepoints (*p<0.05). **Abbreviations:** SOM, somatization; OC, obsession-compulsion; INT, interpersonal sensitivity; DEP, depression; ANX, anxiety; HOS, hostility; PHOB, phobic anxiety; PAR, paranoid ideation; PSY, psychoticism; SLEEP, sleep disorders; GSI, global severity index.

SCL-90: mean scores of SCL-90 subscales in the T0-T2 interval showed a general negative trend, with a more pronounced and statistically significant decrease of PAR score (relative to paranoid ideation; *p* = 0.046) in group 0 and of SOM score (relative to somatization; *p* = 0.001) in group 1 (Fig. **6**).

### 3.3 Correlations Between BIA Parameters and Psychometric Scores

The most frequent positive correlations between BIA parameters and SCL-90 subscales were observed at T0 between the HOS subscale (relative to hostility) and FFM, PA, ICW, BCM, MM, BMR, BCMI and BMI. At T2, similar correlations were observed between the HOS subscale and BMR, MM, BCM, BCMI and BMI. Regarding EDI-2, regression analysis showed a negative correlation between the BU subscale and FM and BMI at T0 and a positive correlation between the IN (relative to inadequacy) subscale and PA, BCM and BCMI at T2 (data not shown).

## 4 DISCUSSION

The results of the present study suggest that treatment of patients with EDs with a standard PNR program associated with AA supplementation induces changes in BIA parameters, suggestive of a halt in the catabolic state (indicated by the increase in the PA value), and the initiation of anabolic processes (reflected in the substantial increase in BCM). These improvements appear to be proportionate to the duration of supplementation, with changes from baseline of BCM, MM and BMR more evident in patients undergoing supplementation at two different dosages for a total of 90 days (group 1) compared with patients undergoing AA supplementation for a total of 45 days (group 0). Simultaneously, the reduction in mean scores of SCL-90 subscales suggests a clear improvement of the psychopathologic state for all patients, whereas the variable changes of individual items of the EDI-2 scores in both subgroups are challenging to interpret.

### 4.1 Bioelectrical Impedance Analysis

Changes of BIA parameters observed in the two groups at each time point may be interpreted as follows: i) the absence of statistically significant differences between groups at T0 confirms the matching of the subjects at baseline; ii) the observation of statistically significant differences of various BIA parameters recorded at different timepoints within each group suggests that treatment, consisting of a standard PNR program and AA supplementation, was effective in improving the nutritional status of enrolled subjects; iii) the observation that statistically significant paired differences of BIA parameters from T0 to T2 were more pronounced in group 1 suggests a further advantage of a more prolonged AA supplementation. Two observations support the possible role of AA supplementation in determining the observed improvements of BIA parameters throughout the study: firstly, in the T0-T1 interval changes were similar in the two groups, which was expected considering that all enrolled patients underwent the same treatment in the first 45 days of the study. In addition, in the T1-T2 interval, statistically significant increases were recorded only in group 1, in which supplementation was prolonged until the end of the study.

**Fig. (7):**
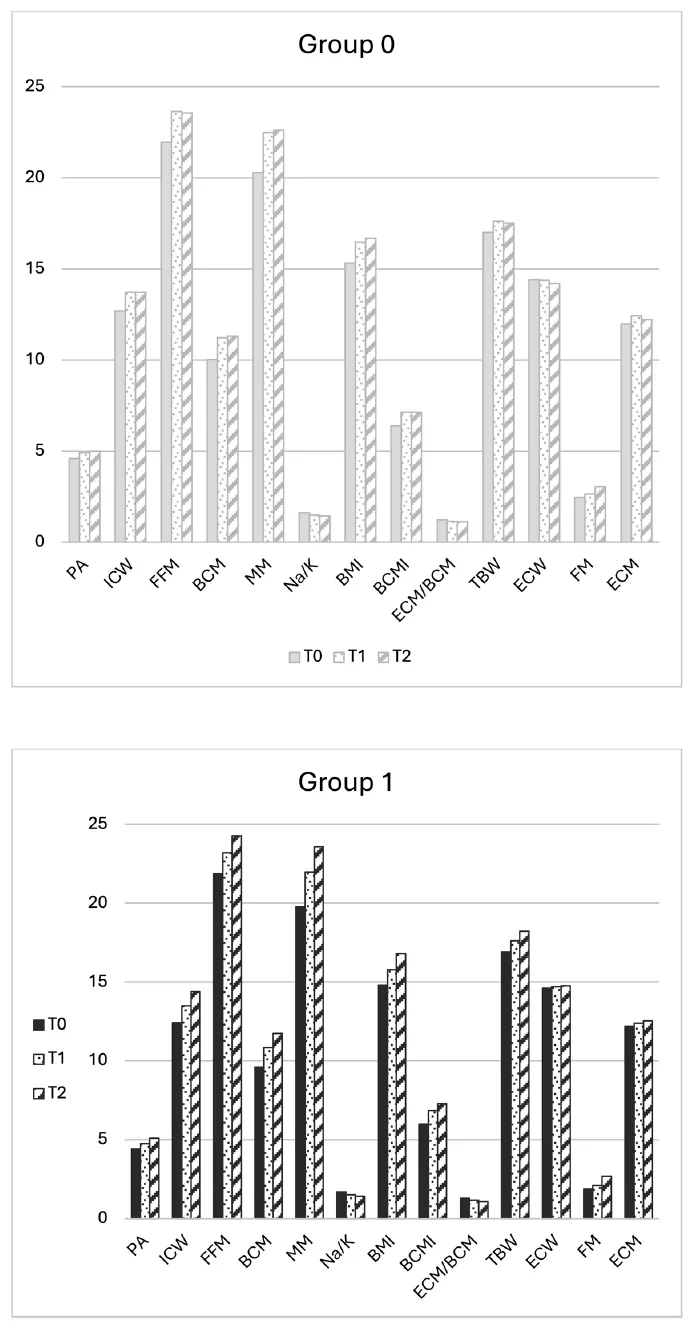
BIA parameters in the two groups at each study timepoint. **Abbreviations:** PA, phase angle; ICW, intracellular water; FFM, fat free mass; BCM, body cell mass; MM, muscle mass; Na/K, Na/K exchange; BCMI, body cell mass index; BMI, body mass index; TBW, total body water; ECW, extracellular water; FM, fat mass; ECM, extracellular mass.

Considering all BIA parameters in the two groups, a general improvement was observed in the T0-T1 interval in all patients, particularly regarding PA, MM, FFM, and BCM. However, whereas in group 0 the increase of these parameters reached a plateau in the first 45 days and remained unchanged in the following 45 days, in group 1 a sustained increase was observed until day 90, which is probably due to the maintenance of the AA supplementation, albeit at a lower dose (Fig. **7**).

These results may be attributed to the combination of a cessation of the severe catabolic state induced by gluconeogenesis resulting from increased energy intake, and the anabolic effect of administered AAs, which may also impact the secretion of growth hormone (GH) and the GHRH/GH/IGF-1 axis [46, 47]. Since body weight increase can revert GH hypersecretion, the subsequent rise in IGF-1 levels may have induced the observed increases in FFM, MM, BCM, BMR and BMI. In this context, a randomized controlled study demonstrated that protein supplementation in patients with NA can increase IGF-1 serum levels [48].

### 4.2 Psychometric Tests and Correlations Between Anthropometric and Psychometric Data

Considering the results of the psychometric tests, the negative trend observed for all SCL-90 subscales in both groups and the correlation observed between the HOS subscale and BIA parameters (BMR, MM, BCM, BCMI and BMI) suggest a potential reduction in the psychopathologic state for all enrolled subjects. This reduction may, to some extent, be attributed to the improvement of the nutritional status and the restoration of the metabolic balance, as suggested by previous studies showing deficiency of various AA in patients with EDs. In this respect, it was demonstrated that tryptophan deficiency can influence serotoninergic transmission in the central nervous system of patients with NA [49] and NB [50], suggesting that such dysregulation may contribute to the pathogenesis of these conditions [51]. It has also been proposed that in NA, lower levels of L-kynurenine and kynurenic acid, deriving from the conversion of tryptophan along the kynurenine pathway, result in diminished stimulation of the aryl hydrocarbon receptor, which could contribute to abnormally low body weight. This evidence supports the notion that NA is an illness in which the deficiency of dietary tryptophan not only reduces the synthesis of serotonin but also hampers kynurenine synthesis, which, in turn, may further contribute to troubled emotions and disturbed eating behaviors [52]. Similarly, tyrosine deficiency following severe dietary restriction may limit the synthesis of noradrenaline [53], whose levels were found to be low in patients with NA [54-56]. Also, methionine deficiency may alter catecholamine metabolism [57, 58], probably associated with low levels of other essential nutrients (folic acid, Vit. B12, B6, riboflavin, zinc, and choline), contributing to neuropsychologic dysfunction causing depression and cognitive impairment often observed in patients with NA. Thiamine (Vit. B1) deficiency, often observed in patients with NA, may contribute to the development of these neuropsychologic symptoms as well [59]. Low levels of valine and isoleucine have also been described in patients with NA [60].

In light of these observations, restoration of normal AA levels through exogenous supplementation may therefore contribute to improvement of both nutritional and neuropsychologic state in patients with NA. For example, tyrosine administration has been proposed as an adjunct treatment through increasing blood tyrosine sufficiently to facilitate brain catecholamine synthesis: in a recent study exploring the pharmacological response to a single, oral tyrosine load in adolescents (aged 12–15 years) with NA and healthy peers, peak tyrosine levels occurred at approximately two to three hours and approached baseline levels by eight hours [61], exceeding the 30–50% increase suggestive of facilitating brain tyrosine changes in rats [62, 63]. Moreover, in patients with NA, prolonged supplementation with tyrosine for 12 weeks was associated with a sustained raised in blood tyrosine over time, a potentially beneficial effect warranting further exploration [61]. Similarly, an animal study aiming to evaluate the effects of glutamine supplementation during refeeding in activity-based anorectic (ABA) mice, showed that glutamine administration is associated with a mTOR-mediated increase in colonic protein synthesis [64]. Such effect is probably due to glutamine ability to regulate protein intestinal metabolism [65] and might be useful to counterbalance the gastrointestinal functional disorders frequently observed in NA patients and often not completely resolved after body weight gain [66]. Finally, the impact of tryptophan or L-kynurenine supplementation on anorexia in animal models and the effects of changes in tryptophan and downstream kynurenines on the clinical progression of NA was explored in various studies: the beneficial effects of tryptophan were shown in a rat model of immobilization stress-induced anorexia, in which tryptophan administration attenuated the impairment of food intake and ameliorated depressive symptoms [67]. The evidence produced so far suggests that the possibility of using essential AA as a supplementary therapy in NA remains open and requires further detailed studies.

## 5 STUDY LIMITATIONS

The primary limitation of this study is the absence of a control group (untreated subjects). Indeed, enrolling a control group without a placebo is difficult in the context of EDs, as administering the same therapy to all patients is essential to ensure adherence. Another limitation of the present study is the short observation period, which, however, reflects the average duration of hospitalization for managing EDs. Although of interest, the results of the present study require confirmation through a larger, randomized, placebo- controlled study of longer duration.

## CONCLUSION

The findings from this study suggest that addition of AA supplementation to a standard PNR program induces significant changes in BIA parameters in patients with EDs. These changes appear to be proportionate to the duration of supplementation, as suggested by more pronounced changes in BIA parameters from baseline to study end observed in patients receiving AA supplementation for the whole duration of the study (90 days), compared to patients who discontinued AA supplementation after 45 days. Although some psychometric indexes positively responded to AA supplementation, the small sample size and the relatively short study duration did not allow for clear observations regarding changes in the severity of psychopathologic state and the degree of symptoms associated with EDs.

## Figures and Tables

**Fig. (1):**
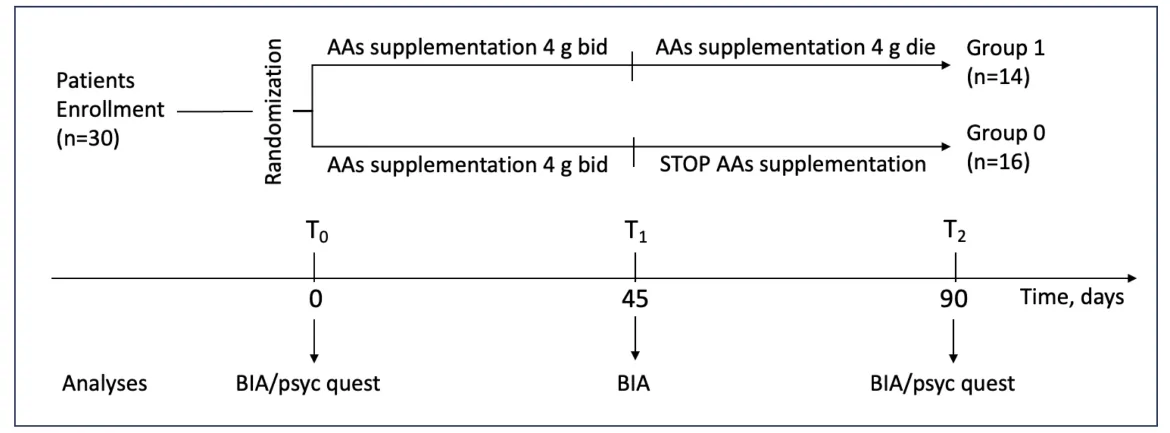
Study design.

## Data Availability

The data and supportive information are available from authors upon request.

## LIST OF ABBREVIATIONS

AAs: Amino Acids
ARFID: Avoidant/Restrictive Food Intake Disorder
BCM: Body Cell Mass
BCMI: Body Cell Mass Index
BED: Binge Eating Disorder
BIA: Bioelectrical Impedance Analysis
BMI: Body Mass Index
BMR: Basal Metabolic Rate
ECM: Extra Cellular Mass
ECM/BCM: Ratio of Extracellular to Body Cell Mass
ECW: Extracellular Water
EDI-2: Eating Disorder Inventory-2
EDs: Eating Disorders
FFM: Fat Free Mass
FM: Fat Mass
GH: Growth Hormone
GHRH: Growth Hormone-Releasing Hormone
ICW: Intracellular Water
IGF-1: Insulin-like Growth Factor 1
MM: Muscle Mass
NA: Nervous Anorexia
Na/K: Sodium/Potassium Exchange Ratio
NB: Nervous Bulimia
PA: Phase Angle
PEM: Protein-Energy Malnutrition
PNR: Psycho-Nutritional Rehabilitation
TBW: Total Body Water
SCL-90: Symptom Checklist-90
ASC: Asceticism
BD: Body Dissatisfaction
BU: Bulimia
DT: Drive for Thinness
IA: Interoceptive Awareness
ID: Interpersonal Distrust
IN: Ineffectiveness
MF: Maturity Fears
P: Perfectionism
SCL-90: Subscales
ANX: Anxiety
DEP: Depression
GSI: Global Severity Index
HOS: Hostility
INT: Interpersonal Sensitivity
OC: Obsession-Compulsion
PAR: Paranoid Ideation
PHOB: Phobic Anxiety
PSY: Psychoticism
SLEEP: Sleep Disorders
SOM: Somatization

## STANDARDS OF REPORTING

STROBE guidelines were followed.
